# Additive Burden of Low Peak Expiratory Flow and Sleep Disturbance on Incident Depressive Symptoms in Later Life: A Four-Cohort Longitudinal Study of 49,201 Participants

**DOI:** 10.3390/healthcare14182951

**Published:** 2026-09-10

**Authors:** Hanlong Li, Hongyu Tan

**Affiliations:** Key Laboratory of Carcinogenesis and Translational Research (Ministry of Education/Beijing), Department of Anesthesiology, Peking University Cancer Hospital & Institute, 52 Fucheng Road, Haidian District, Beijing 100142, China; 2411210627@bjmu.edu.cn

**Keywords:** depressive symptoms, peak expiratory flow, sleep disturbance, lung function, additive interaction, older adults, longitudinal cohort, multi-cohort

## Abstract

Background: Lung function and sleep are each prospectively associated with late-life depressive symptoms, but whether their co-occurrence carries risk beyond the sum of the two components has not been tested across national populations. Methods: We quantified the joint association of low peak expiratory flow (PEF) and sleep disturbance with incident depressive symptoms in four ageing cohorts and formally tested additive and multiplicative interaction. We analysed 49,201 at-risk participants aged 50 to 100 years from the Health and Retirement Study (US), the English Longitudinal Study of Ageing, the China Health and Retirement Longitudinal Study and the Survey of Health, Ageing and Retirement in Europe (16 countries). Low PEF was defined as the lowest quartile within sex and age bands; sleep disturbance used each cohort’s established measure. Incident elevated depressive symptoms were modelled with Poisson generalized estimating equations with robust variance and random-effects meta-analysis. Results: The joint-exposure group had a higher adjusted rate of incident elevated symptoms in every cohort (pooled aRR 1.40, 95% CI 1.28 to 1.52; I^2^ = 41%), corresponding to 7 to 25 additional events per 1000 person-years; component associations were smaller (low PEF only aRR 1.14; sleep disturbance only 1.27). No evidence of interaction was found on either scale, with complete cross-cohort consistency (multiplicative interaction RR 0.95, 0.89 to 1.02; additive RERI −0.03, −0.11 to 0.06; both I^2^ = 0%). Findings persisted in zero-symptom-baseline, sex-stratified, attrition-weighted and competing-risk analyses and were replicated in ELSA when low lung function was defined by spirometric FEV1 (aRR 1.33) or FVC (aRR 1.23) instead of PEF. Conclusions: Co-occurring low PEF and sleep disturbance mark a consistent, additive burden of incident depressive symptoms in later life; combined assessment should be read as simple risk addition, not as a synergistic high-risk phenotype.

## 1. Introduction

Depressive symptoms in later life rarely arrive unannounced. Two candidate warning signs are already measured, cheaply and repeatedly, in community health assessment and population ageing research: peak expiratory flow (PEF), a one-minute respiratory performance test, and self-reported sleep disturbance. Both are prospectively associated with subsequent depressive symptoms [1,2,3,4]. What neither clinicians nor researchers currently know is how to read the two signals together. If a patient or a survey participant presents with both, does the combination signal a risk greater than the sum of its parts—a synergistic high-risk phenotype that might justify intensified monitoring—or simply the arithmetic sum of two independent risks? The answer determines whether combined assessment adds information or merely adds noise.

The pulmonary side of the evidence is prospective and consistent. In 280,032 UK Biobank participants, impaired lung function predicted incident depression [1]; in CHARLS, lower PEF tracked higher depressive-symptom risk [2,5], and physician-reported chronic lung disease predicted incident depressive symptoms across cohorts [6,7]. Depression is also over-represented in chronic obstructive pulmonary disease, where depressive symptoms complicate management and prognosis [8,9,10]. Independently, PEF predicts disability and mortality in community-dwelling older adults, establishing it as a marker of diminished physiological reserve rather than merely of airflow [11].

The sleep side is equally established: insomnia symptoms and abnormal sleep duration prospectively predict depressive symptoms in meta-analyses of longitudinal studies [3,4,12], and recent multi-cohort work shows that sleep features travel across populations as predictors of future depression [13]. Directly at the intersection stands a single CHARLS analysis reporting additive interaction between pulmonary function and sleep duration on depression risk (RERI 0.545, 95% CI 0.053–1.038) [14]. That claim of synergy, derived from one cohort with a three-year follow-up, has never been replicated or tested elsewhere—yet if correct, it would imply that combined PEF–sleep screening might identify a distinct, especially vulnerable group.

Respiratory and sleep pathology also converge in ways that make joint assessment plausible. Sleep-disordered breathing becomes more common with age [15], and obstructive sleep apnoea—a condition that is simultaneously respiratory and sleep-related—is more prevalent in older adults, in whom its consequences are considered more substantial than in younger people [16]. The cohorts studied here ascertained insomnia symptoms and sleep duration rather than sleep-disordered breathing, so apnoea is not part of our exposure definition; but the overlap between respiratory and sleep pathology in later life is a further reason to consider the two domains together rather than in isolation.

We therefore tested the joint association in four population-based ageing cohorts—the Health and Retirement Study (HRS), the English Longitudinal Study of Ageing (ELSA), the China Health and Retirement Longitudinal Study (CHARLS) and the Survey of Health, Ageing and Retirement in Europe (SHARE)—spanning North America, England, China and sixteen European countries [17,18,19,20]. Analyses were prespecified to answer two questions: does the joint PEF–sleep exposure mark elevated incident depressive-symptom risk consistently across cohorts, and does the joint association exceed the sum of the component associations on additive or multiplicative scales?

## 2. Materials and Methods

### 2.1. Study Design and Data Sources

This longitudinal secondary analysis used four population-based cohorts. HRS analyses used the RAND HRS Longitudinal File 2022 (V1) [21]. ELSA analyses used the UK Data Service collection (SN 5050, 50th Edition) and Gateway Harmonized ELSA Version H [22,23]. CHARLS analyses used the public 2011–2020 wave files with the national baseline and Wave 5 guides and Harmonized CHARLS Version D [24,25,26]. SHARE analyses used SHARE Waves 4–9 Release 9.0.0 and Gateway Harmonized SHARE Version G [27,28]. Baselines were HRS wave 8 (2006), ELSA wave 4 (2008), CHARLS wave 1 (2011) and SHARE wave 4 (2011)—the earliest waves at which PEF, sleep and depressive-symptom measures were jointly available. Follow-up depressive-symptom waves were HRS waves 9–16 (2–16 years), ELSA waves 5–10 (2–12 years), CHARLS 2013–2020 (2–9 years) and SHARE waves 5–9 (2–10 years). Reporting follows STROBE [29].

Participants were eligible at age 50–100 years with recorded sex, baseline PEF 30–1000 L/min, an observed sleep exposure, a valid baseline depressive-symptom score and at least one valid follow-up score. Trajectory analyses required at least two follow-up scores. Incident analyses excluded participants above the elevated-symptom threshold at baseline and followed each participant to the first elevated score, last observation or end of follow-up.

### 2.2. Pulmonary Function

PEF was the maximum recorded value from harmonized variables (HRS r8puff; ELSA r4puff; SHARE r4puff, maximum of two blows) and, in CHARLS, the maximum of up to three blows (qb002–qb004) requiring at least two valid attempts with reported full effort (qb005 = 1). PEF was analysed as a field performance measure, not as a spirometric diagnosis of obstruction [11]. Low PEF was defined within each cohort as the lowest quartile within sex and baseline age bands (50–64, 65–74, ≥75 years), an internal definition that absorbs the strong age and sex dependence of raw PEF without imposing a diagnostic cut-off on a non-diagnostic measure. To validate this proxy directly, ELSA sensitivity analyses replaced PEF with nurse-visit spirometric forced expiratory volume in one second (FEV1) and forced vital capacity (FVC) measured at the same wave.

### 2.3. Sleep Disturbance

Sleep disturbance used each cohort’s established measure: in HRS, frequent difficulty in at least one of three insomnia domains (falling asleep, night waking, early waking); in ELSA, frequent difficulty falling asleep; in CHARLS, self-reported nocturnal sleep duration < 6 or >9 h against the 6–9 h reference consistent with adult recommendations [30]; and in SHARE, the EURO-D sleep item (trouble sleeping recently). Because the SHARE sleep item is itself a component of the EURO-D depression scale, SHARE outcomes were computed from the eleven-item EURO-D excluding the sleep item, eliminating exposure–outcome item overlap; the standard twelve-item EURO-D was used in a sensitivity analysis. Sleep constructs differ across cohorts by design; estimates were therefore pooled as a broad sleep-disturbance construct with cohort-specific estimates reported throughout [13].

### 2.4. Joint Exposure

Low PEF and sleep disturbance were crossed into four mutually exclusive groups: neither exposure (reference), low PEF only, sleep disturbance only, and both exposures (joint). This parameterization supplies the directly interpretable group contrasts and the component coefficients required for interaction estimation.

### 2.5. Depressive-Symptom Outcomes

HRS and ELSA used the eight-item Center for Epidemiologic Studies Depression scale (CESD-8, range 0–8), CHARLS the ten-item version (CESD-10, 0–30), and SHARE the EURO-D scale (0–12) [31,32,33]. Elevated symptoms were defined by validated cohort thresholds (CESD-8 ≥ 4; CESD-10 ≥ 10; EURO-D ≥ 4). These instruments quantify symptom burden and do not by themselves diagnose major depressive disorder. The incident outcome was the first follow-up score above threshold among participants below threshold at baseline. For trajectory analyses, follow-up scores were z-standardized within cohort using baseline means and standard deviations, with baseline score as a covariate.

### 2.6. Covariates and Missing Data

Models adjusted for baseline age, sex, education, marital status, smoking, alcohol use, physical activity, body mass index (BMI), self-reported chronic lung disease, a count of six other chronic conditions (hypertension, diabetes, cancer, heart disease, stroke, arthritis), self-rated health, cognition where harmonized, the baseline depressive-symptom score and follow-up time; SHARE models additionally included country fixed effects. Missing categorical covariates retained a missing category; missing BMI was replaced by the cohort median with a missingness indicator; unobserved disease indicators were treated as absent with adjustment for the missing-data count. Physical activity items were administered to a random half-sample in CHARLS waves 1–3 [26], leaving 57.3% missing by design; a corrected multiple-imputation analysis (50 stochastic imputations, Rubin’s rules) predicted missing activity from baseline covariates only—age, sex, education, marital status, smoking, alcohol, BMI, chronic conditions and baseline symptom score—with no follow-up information, avoiding look-ahead leakage.

### 2.7. Statistical Analysis

Incident events were modelled with Poisson generalized estimating equations (GEE; independence working correlation, participant clustering, robust sandwich variance) including linear and quadratic follow-up time, yielding adjusted risk ratios directly and avoiding odds-ratio inflation at common event rates [34,35]. Model-predicted risks generated absolute risk differences (ARD) per 1000 person-years. Multiplicative interaction was the exponentiated low PEF × sleep product term. Additive interaction was the relative excess risk due to interaction (RERI = RR11 − RR10 − RR01 + 1) and the attributable proportion (AP), both on the risk-ratio scale with delta method confidence intervals, reported together as recommended [36]. Trajectories used Gaussian GEE on z-standardized scores with all low PEF × sleep × time lower-order terms. Cohort-specific estimates were combined with DerSimonian–Laird random-effects meta-analysis, with I^2^ summarizing heterogeneity [37,38].

Incident-event data were structured with one observation per participant per follow-up assessment. Each participant contributed observations from baseline to the first assessment above the symptom threshold, the last available assessment, or the end of cohort follow-up, whichever came first; observations after the first event were excluded so that each participant contributed at most one event. We used Poisson GEE rather than a time-to-event model because follow-up assessments occurred at fixed discrete waves shared by all participants within a cohort, so a risk-set formulation was not required, and because the quantity of interest was an adjusted rate ratio rather than a hazard ratio. Poisson GEE with a log link and robust variance estimates rate ratios directly, does not require a proportional-hazards assumption, and accommodates the within-person correlation induced by repeated assessments. The corresponding hazard-ratio formulation was examined separately in Fine–Gray competing-risk models, reported below.

**Figure 1:**
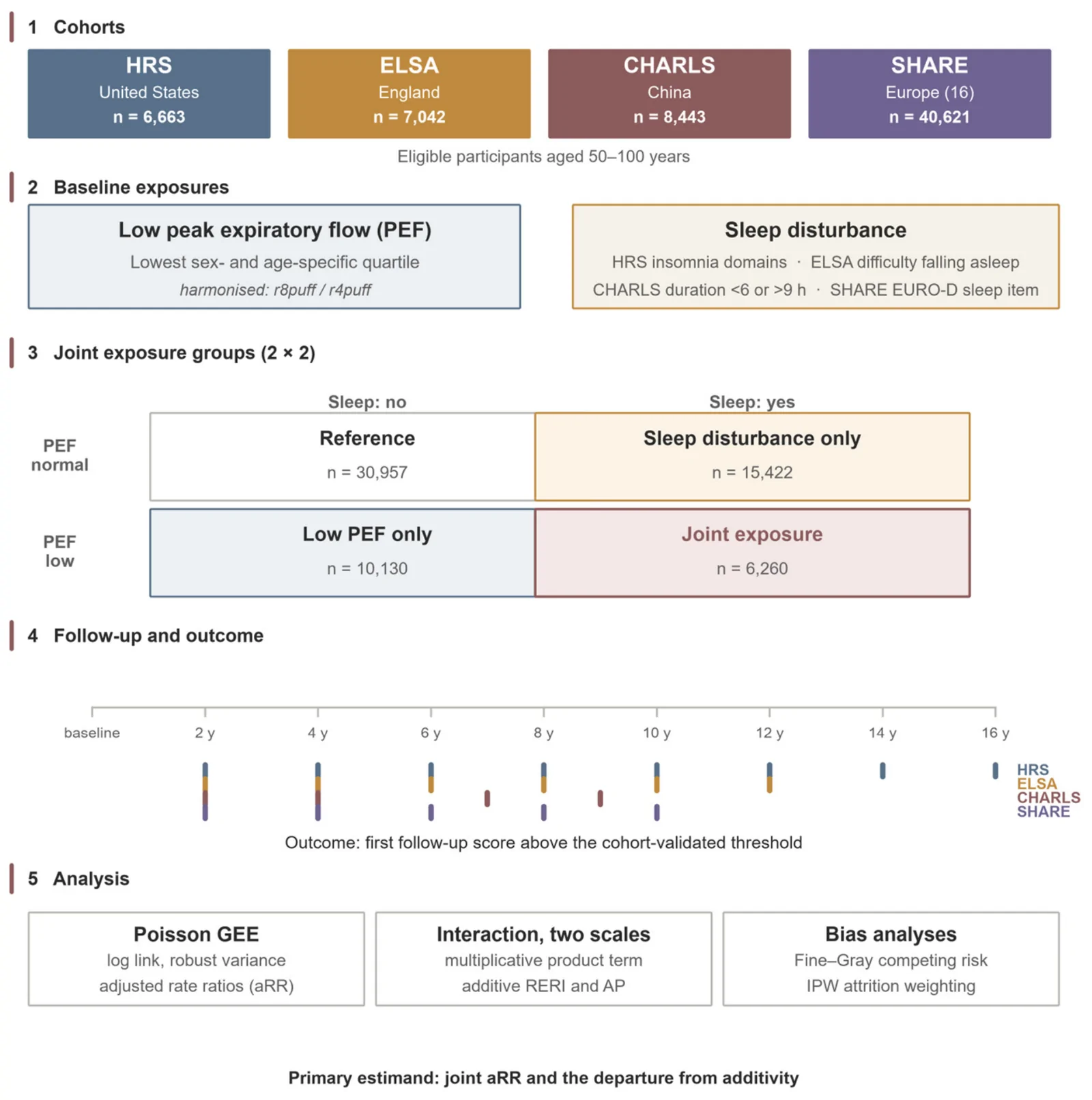
Study design. Baseline assessment of peak expiratory flow and sleep disturbance at each cohort’s baseline wave; classification into four mutually exclusive joint exposure groups; cohort-specific follow-up schedules; and the analytic approach, comprising Poisson generalized estimating equations, interaction on the additive and multiplicative scales, and bias analyses. CESD-8, eight-item Center for Epidemiologic Studies Depression scale; CESD-10, ten-item version; EURO-D, EUROpean Union Depression scale; aRR, adjusted rate ratio; RERI, relative excess risk due to interaction; AP, attributable proportion; IPW, inverse-probability weighting.

Selection and competing-risk bias were addressed explicitly. Baseline characteristics of included participants versus those excluded for absent follow-up were tabulated for every cohort (Table S1). In SHARE, inverse-probability attrition weights (estimated from baseline covariates, truncated at the 1st/99th percentiles) reproduced the primary analysis. In HRS and SHARE, where death dates are available, Fine–Gray subdistribution hazard models treated death as a competing event with inverse-probability-of-censoring weights [39]. Further sensitivity analyses restricted the baseline to completely symptom-free participants (score 0), stratified by sex, and repeated the ELSA incident analysis with FEV1- and FVC-defined low lung function. Analyses used Python 3.13.12 (Python Software Foundation, Wilmington, DE, USA) with pandas 2.3.3, NumPy 2.5.1, SciPy 1.18.0, statsmodels 0.14.6 and lifelines 0.30.3; executable code, variable dictionaries and aggregate targets are provided in the reproducibility package. Two-sided *p* < 0.05 denoted statistical evidence; secondary and interaction findings were interpreted without multiplicity correction. Figure 1 summarises the study design, from cohort enrolment and exposure definition through follow-up to analysis.

## 3. Results

### 3.1. Study Population

**Figure 2:**
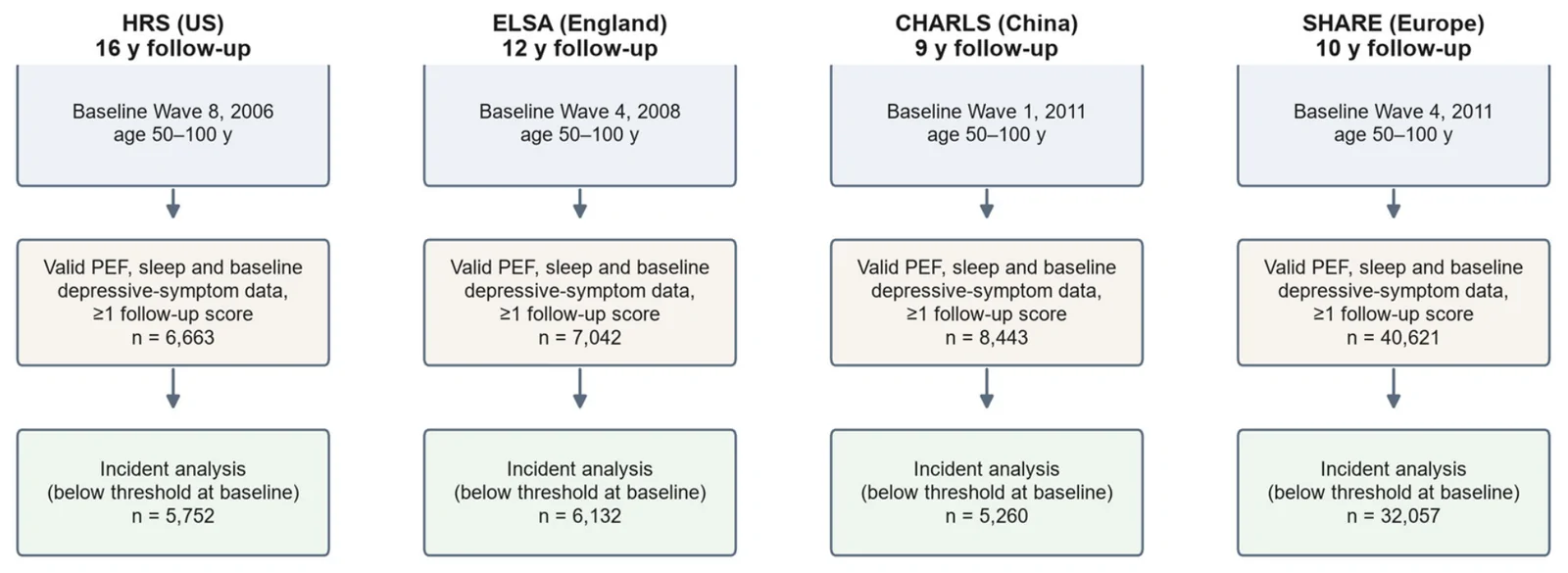
Participant selection and analytic samples in the four cohorts.

The eligible sample comprised 62,769 participants: 6663 in HRS, 7042 in ELSA, 8443 in CHARLS and 40,621 in SHARE (Table 1; Figure 2). The joint-exposure group numbered 6260 participants (695 HRS; 519 ELSA; 889 CHARLS; 4157 SHARE). The at-risk set for incident analysis included 49,201 participants below the elevated-symptom threshold at baseline, contributing 14,562 first elevated-symptom events (1504 HRS; 1427 ELSA; 2399 CHARLS; 9232 SHARE). Trajectory analyses included 52,168 participants with at least two follow-up scores.

Baseline characteristics showed the expected gradient across exposure groups in every cohort (Table 1): the joint group was the oldest and had the lowest PEF, the most chronic disease, and the highest baseline depressive-symptom scores (e.g., mean baseline score in joint versus reference groups: 2.64 vs. 0.88 in HRS; corresponding EURO-D 3.42 vs. 1.63 in SHARE). All models therefore adjusted for baseline symptom level, and incident analyses were restricted to participants below threshold at baseline.

### 3.2. Incident Elevated Depressive Symptoms

**Figure 3:**
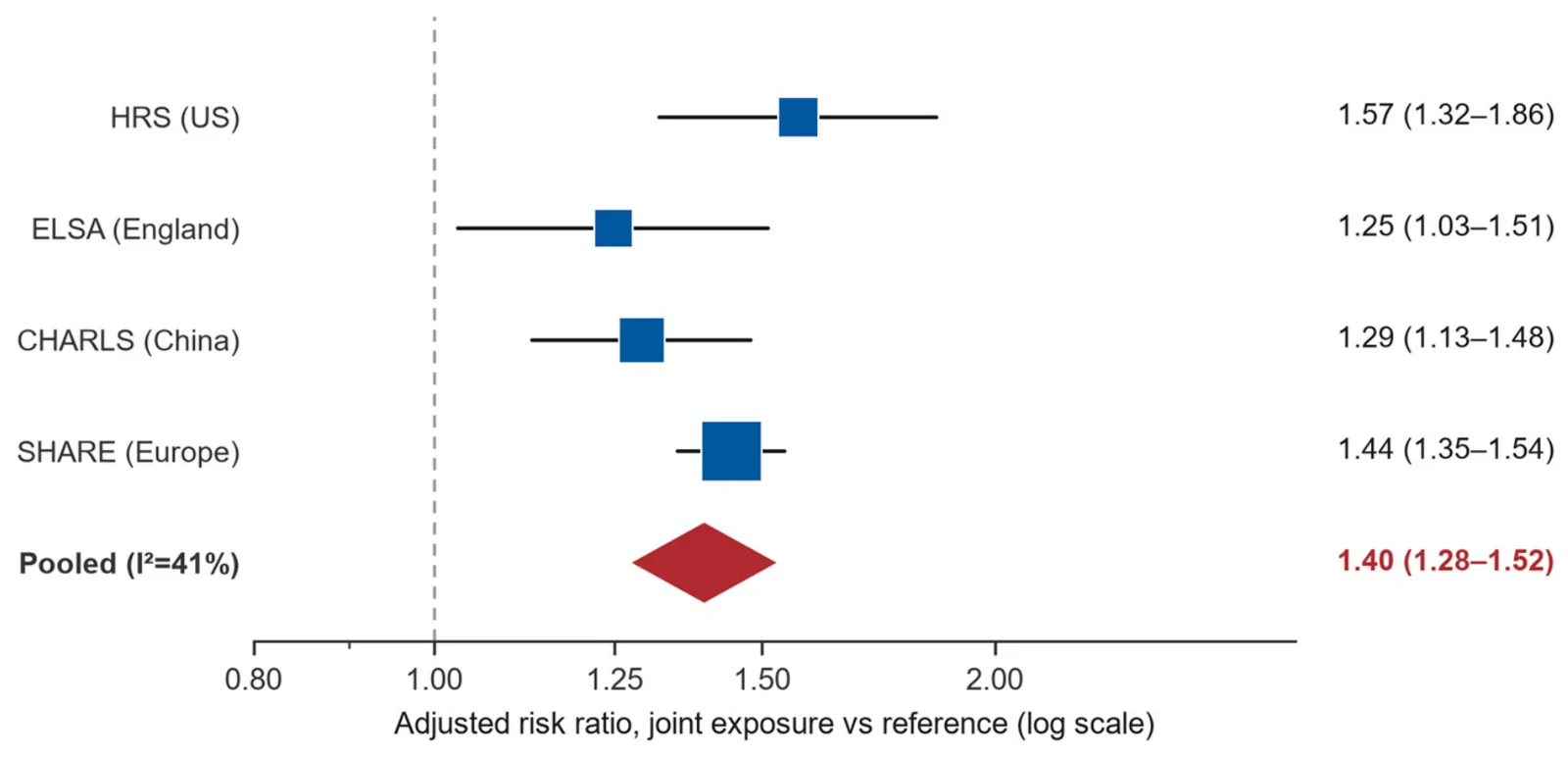
Cohort-specific and random-effects pooled adjusted risk ratios for incident elevated depressive symptoms, joint exposure versus neither exposure. Squares: cohort estimates; diamond: pooled estimate.

The joint-exposure group had a significantly higher adjusted rate of incident elevated symptoms than the reference group in all four cohorts (Figure 3 and Table 2): aRR 1.57 (95% CI 1.32–1.86) in HRS, 1.25 (1.03–1.51) in ELSA, 1.29 (1.13–1.48) in CHARLS and 1.44 (1.35–1.54) in SHARE. The random-effects pooled aRR was 1.40 (1.28–1.52; *p* = 1.0 × 10^−14^; I^2^ = 41%). Component associations were smaller and consistent: low PEF only 1.14 (1.07–1.22; I^2^ = 36%) and sleep disturbance only 1.27 (1.21–1.33; I^2^ = 19%). The joint association translates into 6.6 to 25.0 additional incident events per 1000 person-years across cohorts (HRS 13.4; ELSA 6.6; CHARLS 25.0; SHARE 22.3).

**Figure 4:**
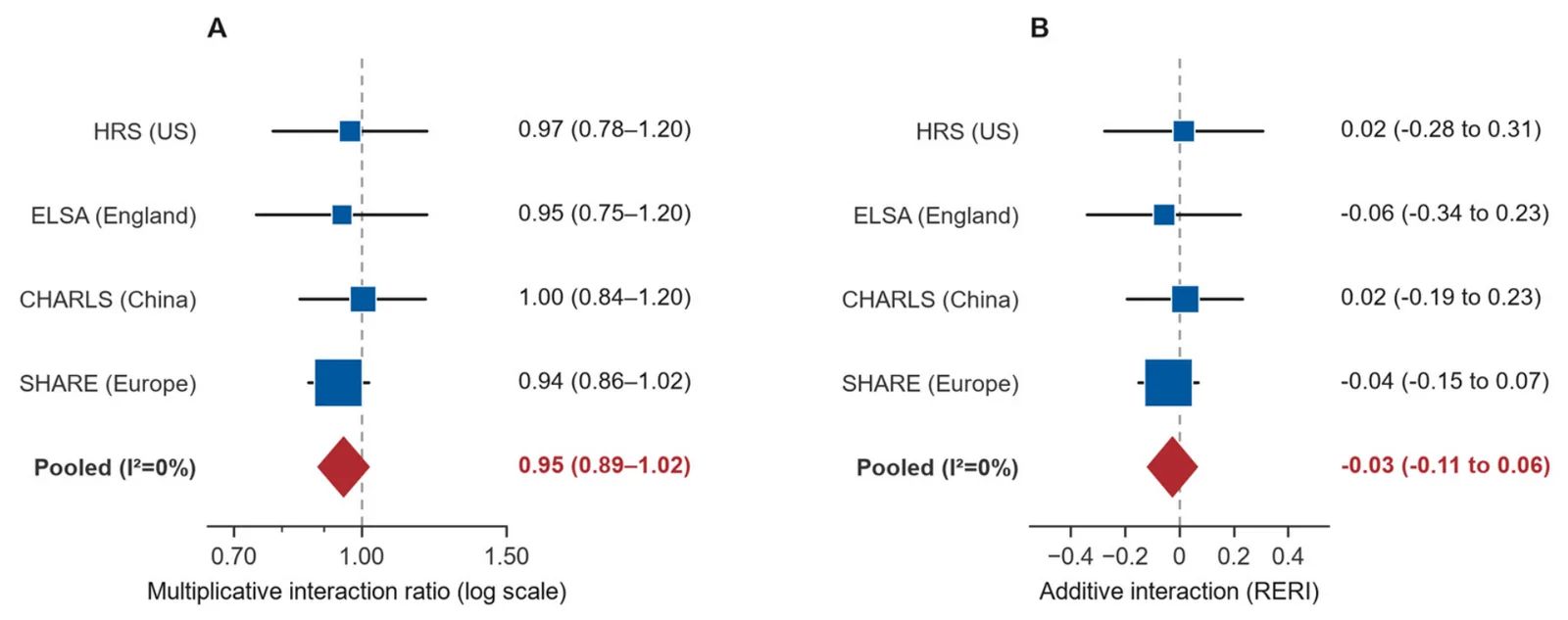
Interaction between low PEF and sleep disturbance on incident elevated depressive symptoms: (**A**) multiplicative interaction ratio; (**B**) additive interaction (RERI). Diamonds: random-effects pooled estimates.

There was no evidence of interaction on either scale, and this absence was itself remarkably consistent (Figure 4). The multiplicative interaction was 0.95 (0.89–1.02; I^2^ = 0%) and the RERI was −0.03 (−0.11 to 0.06; *p* = 0.58; I^2^ = 0%), with every cohort’s additive estimate within ±0.06 of the null. The attributable proportion was −0.01 (−0.04 to 0.02). The joint risk therefore equals the sum of the component risks—additive, not synergistic.

### 3.3. Post-Baseline Symptom Trajectories

**Figure 5:**
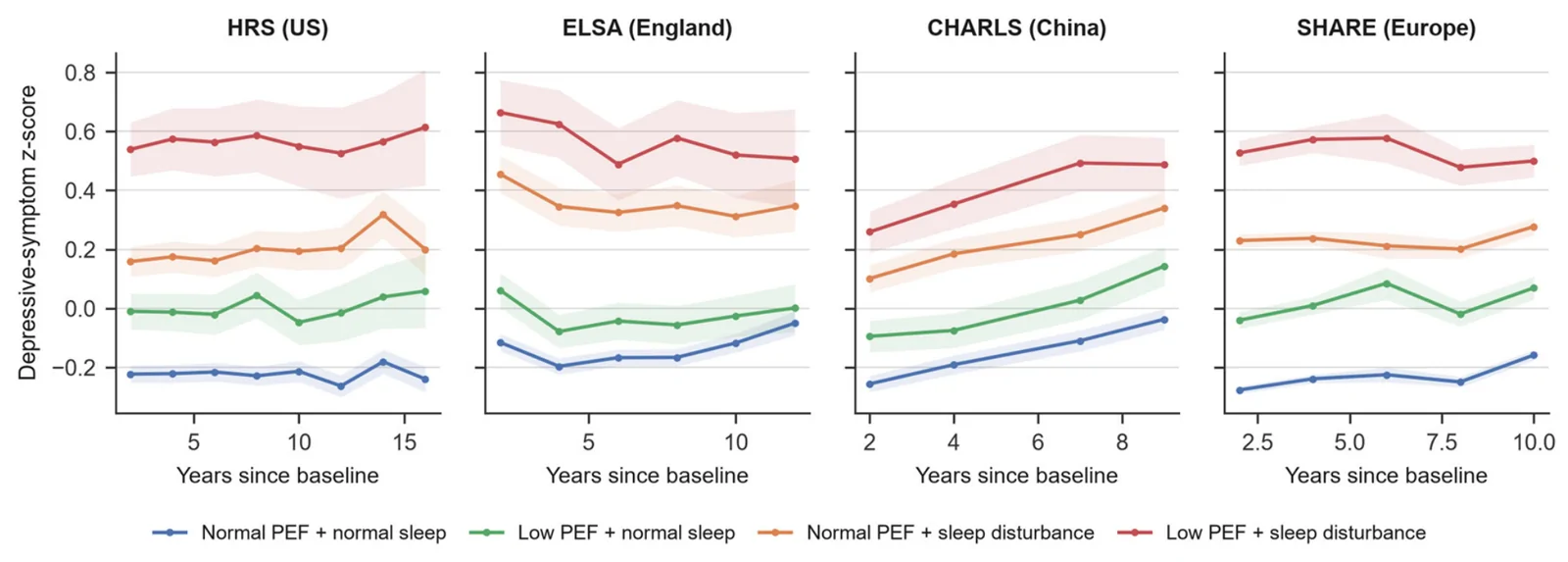
Observed post-baseline depressive-symptom z-scores by four-group exposure profile in each cohort. Shading: 95% confidence intervals.

The joint group also carried a higher sustained symptom burden after baseline: pooled adjusted mean difference 0.180 SD (95% CI 0.142–0.218; *p* = 3.4 × 10^−20^; I^2^ = 46%; Figure 5 and Table 3). The annual slope difference was null (−0.007 SD/year, −0.016 to 0.002; *p* = 0.15), indicating a persistent level difference established early rather than accelerating accumulation.

In CHARLS, symptom levels rose in all four exposure groups across follow-up (mean z-score increase 0.22 to 0.24 between two and nine years), whereas levels were broadly flat in HRS and SHARE and declined slightly in ELSA. The CHARLS rise is unlikely to be exposure-related: the increase was almost identical across the four groups (0.219, 0.240, 0.239 and 0.228 z units in the reference, sleep-disturbance-only, low-PEF-only and joint-exposure groups), and the joint-versus-reference difference remained stable throughout (0.52 to 0.60 z units at successive assessments) with no monotonic widening. A cohort-level secular trend is therefore more plausible than exposure-related divergence and may reflect familiarisation or retest effects on repeated CES-D-10 administration, differential attrition across the Chinese follow-up window, or secular change in sleep and mood among older adults in China between 2011 and 2020. Because the trajectory analysis asks whether exposure groups diverge rather than whether they rise, the CHARLS pattern is compatible with, rather than contrary to, the absence of interaction; it does, however, mean that absolute symptom levels in CHARLS should be read in light of that cohort’s secular context.

### 3.4. Sensitivity Analyses

The joint association strengthened when the baseline was restricted to completely symptom-free participants (pooled aRR 1.74, 1.49–2.03; I^2^ = 0%) and was present in both men (1.37, 1.16–1.63) and women (1.41, 1.32–1.51) (Table 4). It was robust to every bias analysis: Fine–Gray competing-risk models with death as the competing event gave joint subdistribution hazard ratios of 1.72 (1.50–1.98) in HRS and 1.43 (1.37–1.49) in SHARE; attrition-weighted SHARE estimates were unchanged (1.44 to 1.44); the corrected baseline-only multiple imputation of CHARLS physical activity gave 1.29 (1.13–1.48); and the SHARE twelve-item EURO-D specification gave 1.62 (1.52–1.73).

The increase in the joint estimate from 1.40 to 1.74 when the sample was restricted to participants reporting no depressive symptoms at baseline is larger than would be expected from sampling variation alone and deserves interpretation rather than being cited only as a robustness check. Two mechanisms are likely. First, participants with baseline scores above zero but below threshold already carry part of the exposure-related symptom burden; retaining them compresses the exposed–unexposed contrast and biases the estimate towards the null. Second, if low PEF or sleep disturbance is in part a consequence of subclinical depression, analyses that retain symptomatic participants will understate the forward association, whereas the symptom-free restriction removes that admixture. The zero-symptom estimate is thus the least vulnerable to baseline admixture but the most selected towards a healthier subgroup; we report both and treat the primary, less selected estimate as the more conservative summary.

In the direct instrument-validation analysis in ELSA—the one cohort with concurrent PEF, FEV1 and FVC—PEF correlated strongly with FEV1 (Spearman ρ = 0.76; quartile classification κ = 0.45) and FVC (ρ = 0.67) (Table S2). Replacing low PEF with low FEV1 or low FVC reproduced the joint association (FEV1 aRR 1.33, 1.10–1.60; FVC 1.23, 1.01–1.49; PEF 1.25, 1.03–1.51) (Table S3), supporting the interpretation that the inexpensive PEF measurement captures a respiratory risk signal comparable to that identified by spirometry.

Because sleep was operationalised differently across cohorts, we tested whether the CHARLS findings depended on that choice. In CHARLS the two available definitions identified partly different people: sleep duration outside 6–9 h and the CES-D restlessness item agreed for 71.6% of participants, with Cohen’s kappa of 0.37, indicating only moderate agreement. Re-defining CHARLS sleep disturbance by the restlessness item—the construct used by the other three cohorts—and computing outcomes from the nine-item CES-D after removing the sleep item, exactly as the sleep item was removed from the EURO-D in SHARE, left every conclusion unchanged: the joint association was 1.30 (95% CI 1.11–1.52), with no multiplicative interaction (1.06, 0.87–1.30) and no additive interaction (RERI 0.08, −0.16 to 0.32). The near-identity with the primary CHARLS estimate (1.31, 1.14–1.51) indicates that the absence of interaction in CHARLS is not an artefact of how sleep was operationalised in that cohort.

## 4. Discussion

Across four national ageing cohorts and 49,201 at-risk older adults, co-occurring low PEF and sleep disturbance marked a consistent increase in incident depressive symptoms—an excess of 7–25 events per 1000 person-years, or a 40% higher adjusted rate, a burden of a magnitude that warrants prospective evaluation in formal prediction studies. The joint association was compatible with simple additivity in every cohort: the combined risk did not differ measurably from the sum of the two component risks, with no detectable additive or multiplicative interaction and complete cross-cohort agreement (I^2^ = 0% for both interaction measures). Two simple, inexpensive measurements therefore stratify late-life depressive-symptom risk across North American, European and Chinese populations, and they do so by plain addition.

These findings resolve the open question raised by single-cohort evidence. A CHARLS analysis had reported additive interaction between pulmonary function and sleep duration (RERI 0.545) [14], implying a synergistic high-risk phenotype. With four cohorts, more than 14,000 events and prespecified interaction tests on both scales, we find no support for synergy anywhere. The earlier estimate is most parsimoniously read as small-sample interaction instability—a cautionary example of why single-cohort interaction claims require cross-cohort replication before influencing screening practice. The reproducible phenomenon is the additive joint burden, and it is substantial.

The pulmonary measurement itself withstands scrutiny. PEF is effort-dependent and does not replace spirometry; yet in ELSA, where both were measured, low FEV1 and low FVC reproduced the joint association at least as strongly as low PEF, and PEF tracked FEV1 closely (ρ = 0.76). The PEF signal is therefore likely to reflect lung function rather than being an artefact of a convenient instrument—consistent with the established prognostic value of PEF for disability and mortality [11] and with prospective spirometric evidence from UK Biobank [1], although its extension to mental-health outcomes requires confirmation in cohorts with formal spirometry.

Clinically, the additive pattern simplifies rather than complicates practice. Because co-occurrence adds no synergy, no special combined phenotype needs to be learned: a low PEF reading and a positive sleep screen each contribute their own risk, and when both are present their risks simply add in absolute terms, enough additional cases (up to 25 per 1000 person-years) to justify a brief validated depressive-symptom screen in community and primary care settings where both measures are already at hand. The finding equally cautions against over-interpreting combined respiratory–sleep screening tools as markers of a distinct pathological pathway.

Mechanistically, additivity is itself informative: it suggests that diminished respiratory reserve and sleep disturbance act largely additively rather than through a shared amplifying pathway, although the absence of epidemiological interaction does not exclude shared biological mechanisms—on the pulmonary side, spirometric studies have implicated systemic inflammatory and related biomarker pathways [1], alongside reduced physiological reserve [11]; on the sleep side, prospective meta-analyses link insomnia to subsequent affective dysregulation [3,4]. Bidirectional pathways deserve continued attention, as depressive-symptom trajectories themselves predict subsequent respiratory decline [42]; our zero-symptom-baseline analyses, which produced the largest estimates (pooled aRR 1.74), indicate that the forward association reported here is not an artefact of subclinical baseline depression.

The strengths of this analysis are its size and its prespecified design. With 49,201 at-risk participants and 14,562 incident events across four nationally representative cohorts spanning North America, Europe and China, the study had sufficient precision to detect or exclude interaction on two scales, and it is the first to test the PEF–sleep interaction outside a single cohort. Exposures, outcomes and covariate sets were harmonised before analysis; interaction on both the additive and the multiplicative scale was prespecified; the pulmonary exposure was validated against spirometry in the one cohort that measured both; and every conclusion survived restriction to symptom-free participants, sex stratification, attrition weighting, competing-risk modelling and re-definition of the sleep exposure on the construct used by the other cohorts.

Several limitations bound the interpretation. Depressive-symptom scales measure symptom burden, not clinical depression. Sleep constructs differ across cohorts—insomnia domains, sleep duration, and the EURO-D sleep item—so pooled estimates synthesize a broad sleep-disturbance construct rather than a standardized phenotype, although cross-cohort consistency (I^2^ = 0% for interaction) limits concern about construct-driven artefacts. PEF and sleep were measured at baseline only. Residual confounding remains possible despite comprehensive adjustment, attrition weighting and competing-risk analyses, and ELSA and CHARLS lacked death data for competing-risk modelling. Estimates target adjusted within-cohort associations rather than population prevalence, as survey weights were not applied.

Defining low PEF within sex- and age-specific bands was a deliberate choice, and its implications deserve to be stated explicitly. Peak expiratory flow declines steeply and non-linearly with age and differs systematically by sex, so an absolute threshold applied to all cohorts would largely re-measure age and sex rather than respiratory reserve. The cost of the relative definition is that the exposure contrast is specific to each cohort’s own PEF distribution, which limits direct comparison of absolute PEF values across populations and makes the threshold difficult to translate into a single clinical cut-point. Against this, consistency of direction across four populations with different PEF distributions is reassuring rather than circular: the same relative deficit carried a similar association with incident depressive symptoms in each setting, which is what the pooled analysis is intended to test.

Heterogeneity also deserves a more precise reading than the pooled estimates alone convey. For the joint association, I^2^ was 41%, indicating moderate variation in the magnitude of the association across cohorts, as would be expected given differences in sleep measurement, depression scales, follow-up length and population context. For both interaction measures, however, I^2^ was 0%, and every cohort’s confidence interval excluded any materially important interaction. The distinction matters: our claim is not that the magnitude of the joint association is identical everywhere but that the combined risk consistently approximates the sum of its components. The pooled estimate should therefore be read as a summary of direction and approximate magnitude rather than as a single true effect common to all four populations.

## 5. Conclusions

In four national ageing cohorts spanning the US, England, China and sixteen European countries, older adults with both low peak expiratory flow and sleep disturbance experienced a 40% higher adjusted rate of incident elevated depressive symptoms—an excess of 7–25 events per 1000 person-years—with no detectable interaction on the additive or multiplicative scale in any cohort (I^2^ = 0%). The burden is consistent with simple additivity: the joint risk did not differ measurably from the sum of its components, and the finding was validated against spirometric lung function and robust to competing risks, attrition and symptom-free-baseline analyses. Combined respiratory and sleep assessment shows potential as a straightforward additive risk stratification for late-life depressive symptoms but requires prospective evaluation in formal prediction studies before clinical implementation, not as evidence of a synergistic high-risk phenotype.

## Figures and Tables

**Table 1:** Baseline characteristics by joint-exposure group in the four cohorts.

Characteristic	Reference	Low PEF Only	Sleep Disturbance Only	Joint Exposure	SMD†
**HRS (US)**					
*N*	3210	1021	1737	695	—
Participants in incident analysis, *n*	2970	902	1393	487	—
Incident elevated-symptom events, *n*	609	232	475	188	—
Age, years	66.6 (9.7)	68.2 (9.9)	68.0 (9.7)	68.5 (10.1)	0.19
Female, %	56.4	56.4	62.6	61.6	0.11
Current smoker, %	9.0	22.8	9.7	24.3	0.41
Alcohol use, %	56.9	47.4	52.8	42.6	−0.29
BMI, kg/m^2^	29.2 (5.5)	28.8 (6.3)	29.9 (6.1)	29.4 (6.8)	0.03
Chronic lung disease, %	2.8	15.1	6.3	22.0	0.58
Other chronic conditions, count	1.49	1.72	1.84	2.12	—
Baseline depressive-symptom score	0.9 (1.5)	1.3 (1.8)	1.9 (2.1)	2.6 (2.3)	0.90
Baseline PEF, L/min	414.6 (116.2)	232.6 (80.7)	395.1 (108.5)	226.0 (76.4)	−1.92
**ELSA (England)**					
*N*	3909	1252	1362	519	—
Participants in incident analysis, *n*	3627	1118	1045	342	—
Incident elevated-symptom events, *n*	692	259	346	130	—
Age, years	64.7 (8.7)	66.0 (9.4)	65.1 (8.9)	65.4 (9.3)	0.07
Female, %	49.1	48.2	70.4	69.9	0.43
Current smoker, %	7.9	21.8	11.9	27.7	0.52
Alcohol use, %	84.8	78.1	80.6	70.1	−0.35
BMI, kg/m^2^	28.2 (4.9)	27.9 (5.3)	28.9 (5.6)	28.5 (5.9)	0.06
Chronic lung disease, %	12.4	20.5	18.9	26.0	0.34
Other chronic conditions, count	0.95	1.07	1.18	1.30	—
Baseline depressive-symptom score	0.8 (1.5)	1.1 (1.7)	2.1 (2.1)	2.8 (2.4)	0.98
Baseline PEF, L/min	441.1 (129.1)	239.6 (87.6)	391.9 (119.0)	223.7 (78.0)	−2.04
**CHARLS (China)**					
*N*	4149	1373	2032	889	—
Participants in incident analysis, *n*	3041	856	1008	355	—
Incident elevated-symptom events, *n*	1270	403	529	197	—
Age, years	60.7 (7.6)	61.6 (7.1)	61.9 (7.8)	62.8 (7.4)	0.27
Female, %	46.3	48.7	54.3	54.8	0.17
Current smoker, %	33.5	34.8	31.0	31.0	−0.05
Alcohol use, %	35.7	33.0	31.0	32.8	−0.06
BMI, kg/m^2^	23.8 (3.8)	23.1 (3.9)	23.3 (3.8)	22.5 (3.9)	−0.33
Chronic lung disease, %	6.1	17.8	9.3	19.8	0.41
Other chronic conditions, count	0.77	0.81	0.92	0.99	—
Baseline depressive-symptom score	6.9 (5.6)	8.4 (6.2)	10.4 (6.6)	11.8 (6.6)	0.80
Baseline PEF, L/min	350.9 (106.2)	165.4 (58.3)	332.4 (103.3)	156.6 (56.6)	−2.28
**SHARE (Europe)**					
*N*	19,689	6484	10,291	4157	—
Participants in incident analysis, *n*	17,592	5420	6852	2193	—
Incident elevated-symptom events, *n*	3997	1695	2544	996	—
Age, years	64.7 (9.2)	65.8 (9.5)	65.2 (9.4)	66.2 (9.7)	0.16
Female, %	50.4	50.3	67.9	69.8	0.40
Current smoker, %	17.5	24.1	15.7	21.0	0.09
Alcohol use, %	77.4	63.2	69.9	55.0	−0.47
BMI, kg/m^2^	26.8 (4.3)	27.1 (4.7)	27.1 (4.7)	27.5 (5.4)	0.16
Chronic lung disease, %	4.5	9.7	7.5	13.2	0.31
Other chronic conditions, count	0.92	1.08	1.27	1.52	—
Baseline depressive-symptom score	1.5 (1.5)	1.8 (1.8)	2.9 (2.1)	3.5 (2.3)	1.05
Baseline PEF, L/min	428.0 (148.1)	200.3 (79.8)	391.1 (141.5)	183.0 (73.9)	−2.09

Note. Values are mean (SD) unless noted. SMD†: standardized mean difference, joint exposure versus reference group. We used the conventional covariate-balance threshold of |SMD| > 0.10 to flag meaningful between-group differences [40]; this threshold comes from the covariate-balance literature rather than from Cohen’s benchmarks for effect size (0.2, 0.5, 0.8), which were proposed for interpreting standardized effect sizes and are not a general standard for assessing baseline imbalance [41]. Participants in incident analysis and incident elevated-symptom events refer to the at-risk sample after exclusion of participants above the elevated-symptom threshold at baseline. The joint group carried the highest baseline symptom burden in every cohort, motivating baseline-score adjustment and the incident design restricted to participants below the symptom threshold.

**Table 2:** Incident elevated depressive symptoms: adjusted risk ratios, interaction and absolute differences.

Effect	HRS	ELSA	CHARLS	SHARE	Pooled	I^2^, %
Joint exposure vs. reference, aRR	1.57 (1.32–1.86)	1.25 (1.03–1.51)	1.29 (1.13–1.48)	1.44 (1.35–1.54)	1.40 (1.28–1.52)	41
Low PEF only vs. reference, aRR	1.18 (1.01–1.37)	1.06 (0.92–1.22)	1.08 (0.98–1.19)	1.20 (1.13–1.26)	1.14 (1.07–1.22)	36
Sleep disturbance only vs. reference, aRR	1.37 (1.22–1.55)	1.25 (1.09–1.42)	1.19 (1.09–1.31)	1.29 (1.23–1.35)	1.27 (1.21–1.33)	19
Multiplicative interaction, RR	0.97 (0.78–1.20)	0.95 (0.75–1.20)	1.00 (0.84–1.20)	0.94 (0.86–1.02)	0.95 (0.89–1.02)	0
RERI (additive interaction)	0.02 (−0.28 to 0.31)	−0.06 (−0.34 to 0.23)	0.02 (−0.19 to 0.23)	−0.04 (−0.15 to 0.07)	−0.03 (−0.11 to 0.06)	0
AP (attributable proportion)	0.01 (−0.05 to 0.07)	−0.04 (−0.09 to 0.00)	0.02 (−0.01 to 0.05)	−0.03 (−0.05 to −0.01)	−0.01 (−0.04 to 0.02)	62
Absolute risk difference, per 1000 person-years	13.4	6.6	25.0	22.3	—	—

Note. Poisson GEE with robust variance; DerSimonian–Laird random-effects pooling. aRR: adjusted risk ratio; RERI: relative excess risk due to interaction; AP: attributable proportion. Absolute risk differences are derived from model-predicted risks for joint exposure versus reference.

**Table 3:** Post-baseline depressive-symptom trajectories, joint exposure versus reference.

Effect	HRS	ELSA	CHARLS	SHARE	Pooled	I^2^, %
Mean post-baseline symptom level, z	0.254 (0.181 to 0.326)	0.159 (0.085 to 0.232)	0.146 (0.088 to 0.205)	0.176 (0.143 to 0.208)	0.180 (0.142 to 0.218)	46
Annual symptom change, z per year	0.002 (−0.008 to 0.012)	−0.012 (−0.027 to 0.003)	−0.002 (−0.015 to 0.012)	−0.014 (−0.022 to −0.007)	−0.007 (−0.016 to 0.002)	62

Note. Gaussian GEE on within-cohort z-standardized repeated scores, adjusted for baseline score and the full covariate set.

**Table 4:** Sensitivity analyses of the joint-exposure association.

Sensitivity Analysis	HRS	ELSA	CHARLS	SHARE
Zero-symptom baseline, aRR	1.83 (1.24–2.72)	1.77 (1.16–2.71)	2.49 (1.36–4.53)	1.64 (1.35–2.00)
Men, aRR	1.64 (1.22–2.22)	1.23 (0.84–1.81)	1.13 (0.92–1.40)	1.50 (1.33–1.68)
Women, aRR	1.50 (1.22–1.85)	1.25 (1.00–1.56)	1.46 (1.22–1.75)	1.41 (1.30–1.53)
Fine–Gray competing risk of death, SHR	1.72 (1.50–1.98)	—	—	1.43 (1.37–1.49)
CHARLS multiple imputation (baseline-only), aRR	—	—	1.29 (1.13–1.48)	—
SHARE attrition-weighted (IPW), aRR	—	—	—	1.44 (1.35–1.54)
SHARE standard 12-item EURO-D, aRR	—	—	—	1.62 (1.51–1.73)
ELSA low FEV1 instead of low PEF, aRR	—	1.33 (1.10–1.60)	—	—
ELSA low FVC instead of low PEF, aRR	—	1.23 (1.01–1.49)	—	—
CHARLS insomnia-based sleep definition, aRR	—	—	1.30 (1.11–1.52)	—

Note. SHR: subdistribution hazard ratio (death as competing event, at-risk sample). IPW: inverse-probability attrition weighting. Fine–Gray models were not possible for ELSA/CHARLS (death dates unavailable in the licensed files).

## Data Availability

This study used third-party cohort data that the authors are not permitted to redistribute. Qualified researchers can obtain the same source data from the respective providers, subject to registration and the applicable data-use terms: HRS and the RAND HRS Longitudinal File 2022 (V1) from the University of Michigan HRS portal (https://hrsdata.isr.umich.edu/data-products/rand-hrs-longitudinal-file-2022, accessed on 15 July 2026); ELSA (SN 5050) from the UK Data Service via the ELSA data-access page (https://www.elsa-project.ac.uk/data-and-documentation, accessed on 15 July 2026); CHARLS from the National School of Development, Peking University (https://charls.pku.edu.cn, accessed on 15 July 2026); SHARE Release 9.0.0 from SHARE-ERIC (https://share-eric.eu/data/data-access, accessed on 15 July 2026); and the harmonized cohort files from the Gateway to Global Aging Data (https://g2aging.org, accessed on 15 July 2026). The complete analysis code, variable dictionaries, and aggregate result targets needed to reproduce the reported analyses are provided in the accompanying reproducibility package.

## Supplementary Materials

The following supporting information can be downloaded at: https://www.mdpi.com/article/10.3390/healthcare14182951/s1, Note S1: Cohort-specific exposure and outcome definitions; Table S1: Attrition comparison between included participants and those excluded for absence of follow-up; Table S2: Agreement between peak expiratory flow and spirometric lung function (ELSA wave 4); Table S3: ELSA incident analysis by lung-function instrument.
