# Supplementary material for: Additive Burden of Low Peak Expiratory Flow and Sleep Disturbance on Incident Depressive Symptoms in Later Life: A Four-Cohort Longitudinal Study of 49,201 Participants
Source: Healthcare (Basel). 2026 Sep 10;14(18):2951. doi: 10.3390/healthcare14182951 (PMC13606554; doi:10.3390/healthcare14182951)
Supplement: Supplementary file 1 [file healthcare-14-02951-s001.zip › healthcare-4529930-supplementary.pdf]

Supplementary Information

Additive burden of low peak expiratory flow and sleep disturbance on incident depressive symptoms in later life: a four-cohort longitudinal study

Supplementary Note S1. Cohort-specific exposure and outcome definitions

Item	HRS	ELSA	CHARLS	SHARE
PEF	r8puff (max)	r4puff (max)	max of qb002–qb004, ≥2 blows, full effort (qb005=1)	r4puff (max of 2 blows)
Sleep disturbance	frequent difficulty in ≥1 of 3 insomnia domains	frequent difficulty falling asleep (r4fallslp_e 3–4)	nocturnal sleep <6 or >9 h	EURO-D sleep item (r4sleep)
Depressive symptoms	CESD-8, elevated ≥4	CESD-8, elevated ≥4	CESD-10, elevated ≥10	EURO-D ≥4 (11-item excluding sleep item, primary; 12-item sensitivity)
Baseline wave	Wave 8 (2006)	Wave 4 (2008)	Wave 1 (2011)	Wave 4 (2011)
Follow-up waves	9–16 (2008–2022)	5–10 (2010–2020)	2013–2020	5–9 (2013–2022)

Supplementary Table S1. Attrition comparison: included versus excluded for absence of follow-up

Cohort	Group	N	Age	Female, %	Baseline dep. score	PEF	Sleep disturb., %	SRH	Died early, %
SHARE	included	40,621	65.1	56.8	2.09	357	35.6	3.16	0.6
SHARE	excluded_no_followup	9,851	66.7	52.4	2.40	328	36.7	3.39	15.5
ELSA	included	7,042	65.1	54.6	1.29	380	26.7	—	—
ELSA	excluded_no_followup	617	69.4	52.2	1.61	328	29.5	—	—
CHARLS	included	8,443	61.4	49.5	8.52	296	34.6	—	—
CHARLS	excluded_no_followup	809	68.1	48.1	10.04	241	41.5	—	—
HRS	included	12,749	66.7	—	—	—	—	2.73	0.0
HRS	excluded (no 3-wave CES-D)	5,180	73.6	—	—	—	—	3.31	50.1

Note. HRS comparison uses the trajectory-definition requirement (CES-D at waves 8–10). In all cohorts, excluded participants were older, sicker and more depressed; exclusion therefore biases associations toward the null (conservative).

Supplementary Table S2. Agreement between PEF and spirometric lung function (ELSA wave 4, n=6,955)

Spirometric measure	Spearman ρ vs PEF	P	Lowest-quartile κ	N
FEV1	0.76	<0.001	0.45	6,955
FVC	0.67	<0.001	0.32	6,955

Note. κ: Cohen's kappa for lowest-quartile classification (sex × age-band specific).

Supplementary Table S3. ELSA incident analysis by lung-function instrument

Exposure definition	Effect	aRR (95% CI)	P	N	Events
low_PEF (primary)	joint	1.25 (1.03–1.51)	0.0237	6,132	1,427
low_PEF (primary)	low_only	1.06 (0.92–1.22)	0.4444	6,132	1,427
low_PEF (primary)	interaction	0.95 (0.75–1.20)	0.6514	6,132	1,427
low_FEV1	joint	1.33 (1.10–1.60)	0.0029	6,072	1,420
low_FEV1	low_only	1.03 (0.89–1.19)	0.6969	6,072	1,420

low_FEV1	interaction	1.07 (0.85–1.35)	0.5767	6,072	1,420
low_FVC	joint	1.23 (1.01–1.49)	0.0353	6,120	1,424
low_FVC	low_only	1.08 (0.94–1.24)	0.2961	6,120	1,424
low_FVC	interaction	0.90 (0.71–1.14)	0.3933	6,120	1,424

Note. Same Poisson GEE model as the primary analysis; only the definition of low lung function changes.
